# The Effects of Ca^2+^ on Membrane Potential with Altered Function of NALCN and K2P Channels

**DOI:** 10.3390/membranes16090298

**Published:** 2026-09-11

**Authors:** Youngwoo Kim, Jiwoo Kim, Robin L. Cooper

**Affiliations:** 1 Department of Biology, University of Kentucky, Lexington, KY 40506, USA; ywkim17@outlook.com (Y.K.); jiwoo.kim541@topper.wku.edu (J.K.); 2 The Gatton Academy, 1906 College Heights Blvd. #71031, Bowling Green, KY 42101, USA; 3 Model Laboratory School, 521 Lancaster Ave, Richmond, KY 40475, USA

**Keywords:** calcium, K2P, muscle, NALCN, potential

## Abstract

Cells exhibit a membrane potential due to the differential distribution of ions and the density of channels, pumps, and exchangers. It is known in larval *Drosophila* that the membrane potential gravitates towards the equilibrium potential of K^+^ due to the high density of K2P channels. Higher extracellular Ca^2+^ ([Ca^2+^]_o_) tends to drive the resting membrane potential to a more negative state, while lowering it has an opposite effect. The expression of the K2P channel and NALCN was altered genetically to determine the sensitivity to changes in [Ca^2+^]_o_, and computational simulations using the theoretical Goldman-Hodgkin-Katz equation allowed estimating changes in ion (Na^+^) permeability due to altered function of the NALCN. Increasing [Ca^2+^]_o_ hyperpolarized the membrane potential, and decreasing [Ca^2+^]_o_ depolarized it, likely because Ca^2+^ ions block NALCN. An accessory protein to NALCN and NALCN itself were targeted by RNAi. Overexpression of K2P channels and decreased NALCN function reduced the effect of altered [Ca^2+^]_o_ on membrane potential. Given the limited understanding of how altered membrane potentials affect cells, this study provides a foundation for future investigations into how cells respond to variations in [Ca^2+^]_o_ under altered K2P and NALCN expression.

## 1. Introduction

The electrical potential across the membrane of cells and organelles depends on the electrochemical gradient of various ions. The electrochemical gradient encompasses two parts: a chemical gradient, which refers to the diffusive movement of ions, and an electrical gradient, which refers to the difference in electrical charge across the membrane. This electrical charge is influenced by the differential distribution of ions, which itself is due to various factors, such as leak channels, pumps, exchangers, and even voltage-gated ion channels. The degree of the resting membrane potential (RP) can be large and generally ranges from negative 100 mV to negative 20 mV depending on the cell or organelle type [1,2]. The charge difference across the membrane is also partly due to charges on the glycoproteins and molecules on the surfaces of the membrane [3]. In a resting steady state, the leak channels for ions, ionic pumps, and exchangers are the major contributors maintaining an electrical gradient. Inhibiting a pump, such as the Na^+^-K^+^ ATPase, generally leads to depolarization, and depending on the cell type, other pumps and exchangers may also contribute to the potential. A cell at rest is sensitive to cytoplasmic Ca^2+^ and can rapidly pump Ca^2+^ out of the cell via the plasma membrane calcium ATPase pump (PMCA). A common exchanger among cells is the sodium-calcium exchanger (NCX), which is bidirectional, exchanging Ca^2+^ efflux for an influx of Na^+^ when considering that Na^+^ is generally at a higher concentration in the extracellular fluid (ECF).

Most cells studied show that membrane potential behaves like a K^+^ electrode, detecting subtle changes in the concentration of free K^+^ ions on either side of the membrane. Many cells in various animal models will depolarize in a linear fashion as extracellular K^+^ concentration (i.e., [K^+^]_o_) is increased. If intracellular K^+^ (i.e., [K^+^]_i_) is decreased, a similar trend will occur. If [K^+^]_i_ increases, the membrane potential will become more negative. There are exceptions for regions of cells and particular sensory cells [4,5]. For many cells, the resting membrane potential (RP) is also dependent on a small leak of Na^+^ influx, thus driving the membrane in a more depolarized state away from the equilibrium potential for the K^+^ ion alone (E_K_). It is now established that the leak of K^+^ ions is due to a family of K^+^ channels referred to as K2P channels (i.e., two-pore domain K^+^ channel) [6,7]. The Na^+^ leak is due to NALCN (i.e., Na^+^ leak channel) proteins [8].

The membrane potential can loosely be estimated by the Goldman-Hodgkin-Katz (G-H-K or GHK) equation. This is also referred to as the constant field equation, considering the field potential across the membrane is constant [2]. The G-H-K equation considers the permeability of multiple ions (K^+^, Na^+^, Ca ^2+^, and Cl^−^) and their gradients across a cell membrane [9,10,11,12,13,14,15] but does not account individually for pumps and exchangers at various RP values.

The larval *Drosophila* body wall muscles (i.e., skeletal muscles) allow one to examine the membrane potential with ease by the use of fine-tip glass microelectrodes while changing the bathing media to different ionic concentrations. In addition, genetic strains of *Drosophila* can be used to alter the expression of NALCN or the K2P channels. There are 11 known K2P subtypes known to be present in the *Drosophila* genome; however, their expression profiles in the various tissues are unknown [16,17]. Previous studies have overexpressed the *Drosophila* ORK1–K2P channel subtype in muscle, which resulted in hyperpolarization of the resting membrane potential (RP) [18]. In these past studies, the RP tended to remain closer to the equilibrium potential (E_K_) because a high density of K2P channels allowed K^+^ ions to flow down their concentration gradient. Thus, the RP is less susceptible to Na^+^ leak by the NALCN. In addition, compromising NALCN expression or function is predicted to drive the RP even more strongly toward E_K_. It is known for larval *Drosophila* muscle that a higher extracellular Ca^2+^ (i.e., [Ca^2+^]_o_) tends to drive the RP to a more negative state, while lowering [Ca^2+^]_o_ has an opposite effect. This is assumed to be due to extracellular Ca^2+^ blocking NALCN channel function [19,20,21].

The ability of the NALCN channel to properly function is related to the presence of the associated accessory proteins in the NALCN protein complex. When the protein Mid1 (i.e., “mating-induced death” phenotype), also known as Nif-1 in *Drosophila*, was reduced, so was the expression of the NALCN channels [21,22,23,24]. Mid1 was initially identified in yeast, and if expression was altered, the function of NALCN channels in fungi and animals was compromised [25]. With the use of the RNAi strain for the coded gene *na* (i.e., NA-narrow abdomen) in *Drosophila,* it was demonstrated that there was a reduction in the expression of NALCN [8]. The ease of genetic manipulation and the alteration in expression of NA and Mid-1(Nif-1) in specific tissues, such as body wall muscles, in the *Drosophila* model allow the assessment of physiological measures of membrane potential. The use of larval *Drosophila* muscle also reduces the variability to account for Na^+^ leak through voltage-gated Na^+^ channels, as it appears the larval muscle does not express voltage-gated Na^+^ channels [26,27]. In this study, we targeted both NA and Mid1 using mRNAi strains to examine their effect on RP. In addition, the [Ca^2+^]_o_ was changed from 1 mM in the standard saline to either 0 mM or 5 mM while recording the RP. Thirdly, to examine the effect on RP when the ORK1–K2P channel is overexpressed would address whether RP would be less sensitive to changes in [Ca^2+^]_o_.

Since only one variable in the G-H-K equation is being altered in this investigation by comprising the NALCN, one can estimate the change in the permeability of Na^+^ (i.e., P_Na_) to account for the differences in the RP. We did not directly measure Na^+^ permeability but estimated it to fit the GHK from the measured membrane potentials. With the new homeostatic state of the RP in the overexpressing strain of the ORK1–K2P channel, an adjusted P_K_ could then be used to determine again how much P_Na_ would need to be altered to account for the changes in RP with the different Na^+^ flux due to increasing and decreasing the block of the NALCN channels by altering the [Ca^2+^]_o_. Simulations provide an estimation of the changes that would need to occur to account for the changes in RP due to differences in [Ca^2+^]_o_ and varied expression of NALCN and K2P channels in this model preparation. This serves as a foundational study for future investigations in addressing how other cells may respond in altered states of expression and variation in [Ca^2+^]_o_.

In pathological conditions, such as cancer, as well as in other diseased states, K2P channel expression is known to be altered [28,29]. The effect of altered [Ca^2+^]_o_ on the RP of such cells has not been addressed. In addition, there are diseased states related to NALCN dysfunction [30], and such states have not been addressed with altered [Ca^2+^]_o_ on the RP. Even without known abnormalities in the expression or dysfunction of K2P and NALCN proteins, hypo- and hypercalcemia are known to have physiological effects on the excitability of neurons (i.e., Chvostek’s sign and Trousseau’s sign) [31,32,33]. Such a comorbidity of abnormal [Ca^2+^]_o_ and a diseased state affecting K2P or NALCN channels has not been addressed or reported as far as we could determine. Thus, if such pathological conditions are identified, one may have a better understanding of the consequences that could be related to the membrane potential and cellular function.

## 2. Materials and Methods

The methodological approaches used are very similar to the procedures described in Hana et al. [20] for examining the effects of Mg^2+^ on the membrane potential, as well as a previous study that addressed the acute effects of lipopolysaccharides (LPS) on membrane potential with various ionic compositions of saline [18]. In brief, the same procedures are described below, along with the differences used in this study.

### 2.1. Drosophila Strains

Overexpression of the ORK1 protein (a K2P channel) in the larval muscle m6m7 was achieved by crossing homozygous males of BG487 (BDSC stock # 51634) and UAS-ORK1 (BDSC stock # 6586). We referred to the F1 as M6M7>ORK1. We used two RNAi lines to affect NALCN expression. Control overexpression of non-conducting K2P channels was obtained by crossing BG487 males with female virgins bearing a non-conducting ORK1 transgene (BDSC stock # 6587); these progenies were referred to as M6M7>ORK1-NC. This line also featured GFP co-expression to confirm successful crossing into the specific m6m7 muscles. Female virgins of mid1 RNAi (BDSC stock # 36754) were crossed with the BG487 M6M7-Gal4 males and referred to as M6M7>MID-RNAi. Also, female virgins of NA-RNAi (BDSC stock # 25808) were crossed with BG487 m6m7-Gal4 males and referred to as M6M7>NA-RNAi. The UAS-NA-RNAi, UAS-Mid1-RNAi, UAS-ORK1, and M6M7-Gal4 were used as parental controls for this study. Larval body wall muscles 6/7 feature an anteroposterior gradient pattern of M6M7-Gal4 expression, allowing BG487 to drive UAS-gene expression in those muscles selectively [34,35]. We maintained larvae at room temperature (~21 °C) in vials partially filled with a cornmeal-agar-dextrose-yeast medium. Only early 3rd instars were used for physiological studies.

### 2.2. Dissection and Physiology

We described the dissection procedures and electrophysiological recording methods in a previous study [19]. We used the m6 muscle in segment two throughout these studies to monitor transmembrane potentials. To expose the muscle, the brain was cut from the segmental nerve roots. To perform this without damaging the m6 muscle, the brain was lifted up via the gastrointestinal tract and draped over the dissection pin as illustrated earlier (Figure 1B in [36]). Only early third-instar larvae were used. The fine ends of sharp intracellular electrodes (30 to 40 megaOhm resistance) were filled with 3 M KCl, then inserted into muscle. An AxonClamp 2B (Molecular Devices, Sunnyvale, CA, USA) amplifier, 1 X LU head stage, and LabChart 7.0 (ADInstruments, Colorado Springs, CO 80907, USA) were used for data collection and analysis.

A modified hemolymph-like 3 (HL3) [37,38] was used as the saline for dissection. The saline consisted of (in mmol/L) 70 NaCl, 5 KCl, 10 MgCl_2_, 10 NaHCO_3_, 5 trehalose, 115 sucrose, 25 N, N-bis(2-hydroxyethyl)-2-aminoethane sulfonic acid (BES), and pH 7.2. CaCl_2_ used for the dissections and starting saline for recordings was 1 mM CaCl_2_. Throughout the experiments, the concentration of CaCl_2_ was varied from 0 to 5.0 mM. The preparations were incubated in the saline solutions for 3 min. Following 3 min, the membrane potential was measured for index purposes. If the membrane potential did not remain stable during the initial saline exposure for 3 min, the analogous muscle fiber on the contralateral side was tried. If neither muscle provided a stable initial recording, then a new preparation was used. If the preparation pulled loose from the dissection pins, which could happen in 0 mM [Ca^2+^]_o_ saline, the preparation was discarded, and a new preparation was obtained.

### 2.3. Simulations

To program the simulation, a Python-integrated environment (i.e., Python extension of the VSCode platform) and two GitHub codes (https://github.com/ywkim17/Educational_Book/blob/main/Nernst.py (accessed on 10 August 2026) and https://github.com/ywkim17/Educational_Book/blob/main/GHK.py) (accessed on 10 August 2026) were used. The codes named “Nernst.py” and “GHK.py” (as detailed online, [39]) were used via the VSCode software. The coding is provided in Appendix A.

### 2.4. Statistical Analysis

Data are quantified as mean (±SEM-standard error of the mean). Paired *t*-tests were conducted to compare responses before and after solution exchange. The Shapiro-Wilk test was conducted to check for normality. When data were not assumed to be normal, the Wilcoxon rank-sum test, the non-parametric version of the *t*-test, was used. The Wilcoxon rank-sum test does not assume normality of the population, making it suitable for ordinal or continuous data. The test compares the ranks of the data points from both samples to determine if they come from the same population. The main assumptions for this test are that the data are at the ordinal or continuous level and that observations from both samples are independent. These conditions were met when this test was used. Since sample sizes were consistent, two-way analysis of variance (ANOVA) with Tukey’s method was conducted to compare different larval strains. The Sigma Stat (version 4, Systat Software Inc., San Jose, CA, USA) software was used for analysis. A *p*-value of *p* < 0.05 was considered statistically significant.

## 3. Results

Initially, a series of recordings in individual preparations of the membrane potential was used while exchanging the saline with various concentrations of [Ca^2+^]_o_ as follows: 1-0-1-5-1 mM. However, it was difficult to maintain good intracellular recordings either due to disturbance by the saline change for the fine-tipped glass microelectrode in the muscle fiber or due to the muscle twitching when switched to a 0 mM [Ca^2+^]_o_ saline. Although we obtained at least six good recordings, we changed the protocol to use only single preparations for saline exchanges with concentrations of 1-0-1 or 1-5-1 mM [Ca^2+^]_o_. The neuromuscular junctions (NMJs), when exposed to 0 mM [Ca^2+^]_o_ saline, would show random excitatory junction potentials on occasion with the initial change and then stop. This was likely due to the segmental nerves spontaneously being excited as nerves become hyperexcitable in low [Ca^2+^]_o_ and due to some residual Ca^2+^ around the subsynaptic reticulum of the NMJs until it was flushed away with the exchange of the bathing media. Preparations in which it appeared the muscle fiber was damaged by the twitching and tearing of the membrane with the microelectrode were discarded. Such twitching was not observed when switching from the 1 to 5 mM [Ca^2+^]_o_ salines.

### 3.1. Parentals of the Non-Conducting and Conducting K2P Channel Strain: Parental UAS-ORK1-NC, UAS-ORK1 and Parental M6M7-Gal4

**Figure 1:**
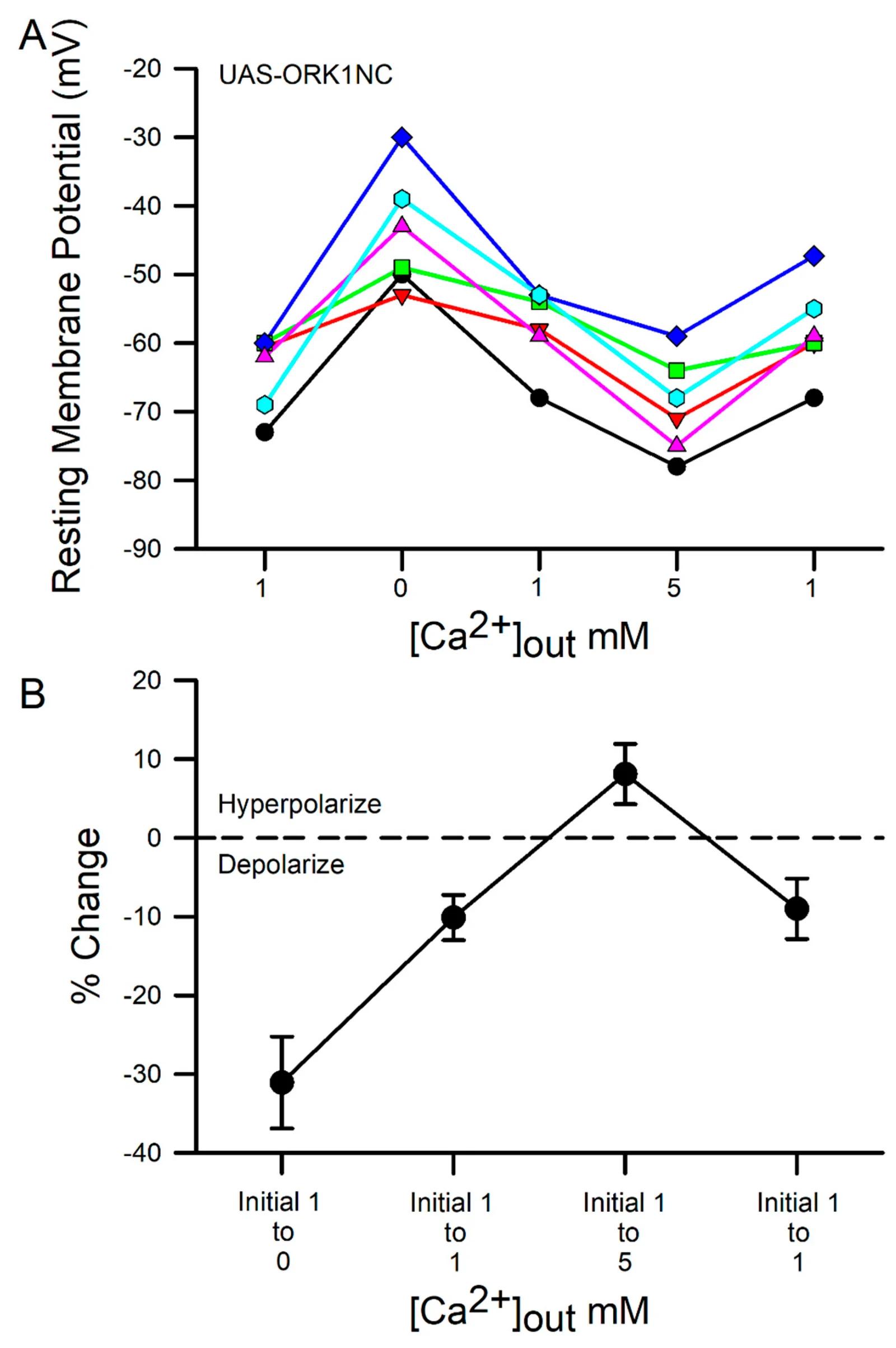
The effect on membrane potential for the parental strain UAS-ORK1-NC with serial changes in [Ca^2+^]_o_. (**A**) Membrane potential for six individual preparations bathed in 1 mM [Ca^2+^]_o_, then switched to 0 mM and back to 1 mM, followed by 5 mM and back to 1 mM, with intracellular electrode recording. Each line represents an individual preparation. (**B**) The average (±SEM) of the percent change in the membrane from the initial values bathed in 1 mM [Ca^2+^]_o_ to each condition in the series of bath changes in [Ca^2+^]_o_. Note that, even when replaced with the bathing medium back to 1 mM [Ca^2+^]_o_, the preparations still showed a depolarization.

One of the strains used as a control for overexpression of protein in the muscle fiber was the M6M7>ORK1-NC. This strain is independent of K2P conductance and is used to mimic the overexpression of ORK1 at a protein level, to match any potential change in cellular physiology or channel-associated signaling. Because ORK1-NC is a non-functional K2P channel, it was not expected to produce a more negative RP under resting conditions at 1 mM [Ca^2+^]_o_. As a control for the F1 strain (i.e., M6M7>ORK1-NC), the parental strains were also examined (i.e., UAS-ORK1-NC and M6M7-GAL4). With the UAS-ORK1-NC strain, it was possible to collect enough good recordings to complete the full series of saline exchanges as 1-0-1-5-1 (Figure 1A). Each of the six individual preparations showed the same trend with depolarization in 0 mM and a subsequent hyperpolarization from the 1 mM [Ca^2+^]_o_ exchanged after exposure to 0 mM (*p* < 0.05, paired *t*-test). However, the return to 1 mM after 0 mM did not allow the muscles to completely regain the RP to the initial values, even though the incubation was allowed for three minutes. Thus, the RP values during the second exposure to 1 mM remained depolarized. This could be due to slight muscle damage due to the low [Ca^2+^]_o_ exposure and brief twitching of the muscle. Upon switching the saline to 5 mM [Ca^2+^]_o_, the RP showed a pronounced hyperpolarization in all six preparations (Figure 1; *p* < 0.05, paired *t*-test); however, not to the initial 1 mM [Ca^2+^]_o_. The percent change in the RP relative to the initial values of 1 mM [Ca^2+^]_o_ is shown (Figure 1B). The percent change helped normalize the varied initial RPs among preparations. Thus, the protocol was changed, as mentioned above, to 1-0-1 and 1-5-1 to have more reliable data on the effects of changing the [Ca^2+^]_o_ for 1-0-1 and 1-5-1 on the RP.

**Figure 2:**
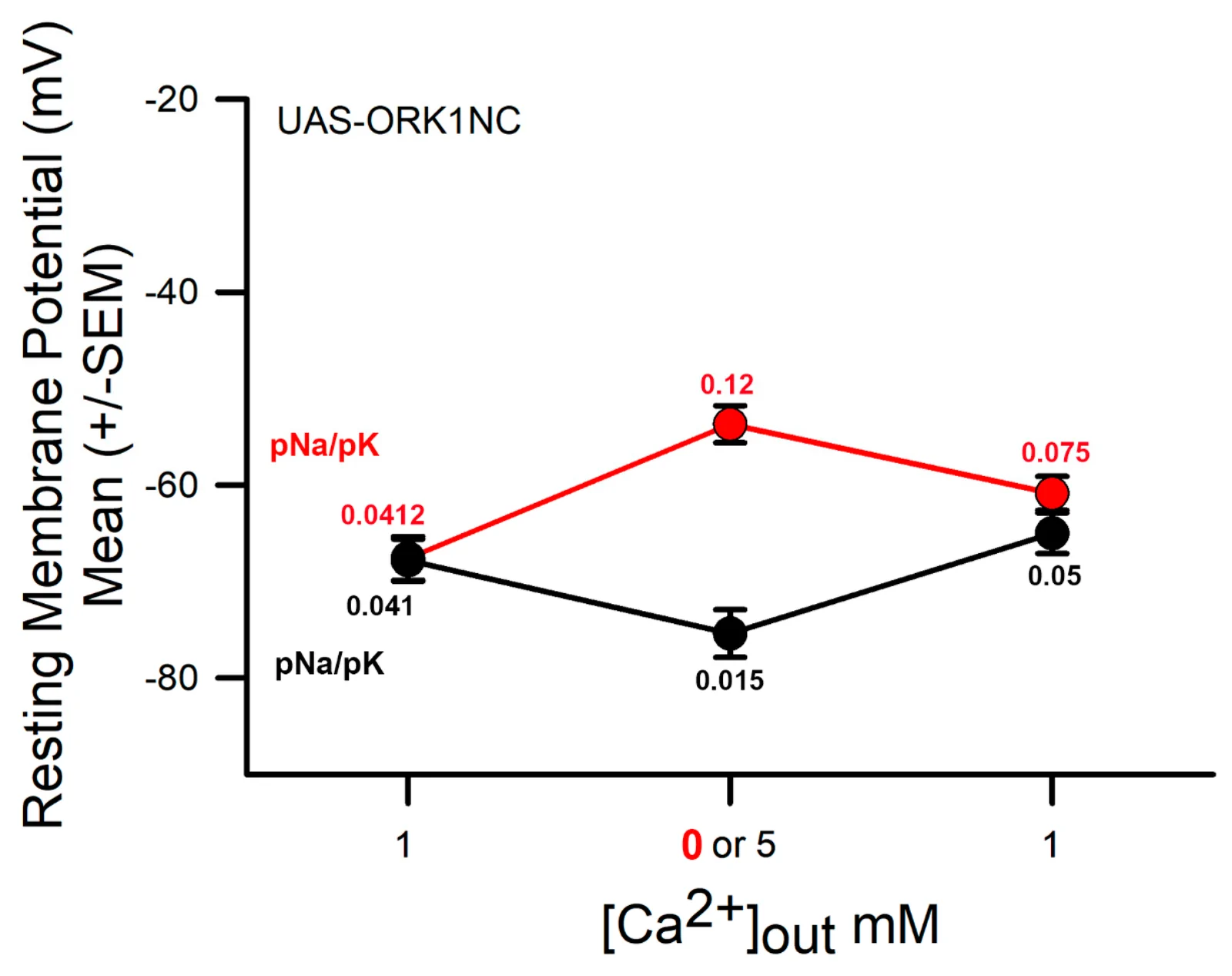
The effect on membrane potential for the parental strain UAS-ORK1-NC with changes from 1 mM to 0 or 5 mM and back to 1 mM [Ca^2+^]_o_. The average (±SEM) of the change for 10 individual preparations. *p* < 0.05, paired *t*-test, n = 10 for each of the paradigms 0 mM-1 mM or 0 mM to 5 mM. The estimated P_Na_/P_K_ values are presented for each condition. These estimates did not alter the PK values.

Using the same parental strain UAS-ORK1-NC, another series of experiments was conducted with individual preparations for each paradigm 1-0-1 and 1-5-1 [Ca^2+^]_o_. The same trends were observed but with more reliable data for the effect of exchanging the bathing media from 1 to 5 mM [Ca^2+^]_o_ (Figure 2). The change to 0 mM [Ca^2+^]_o_ produced a significant depolarization, and the change to 5 mM [Ca^2+^]_o_ produced a significant hyperpolarization (*p* < 0.05, paired *t*-test, n = 10 for both paradigms). The estimated P_Na_/P_K_ values indicate how much P_Na_ would approximately need to change for each concentration of Ca^2+^ to either block or relieve the NALCN block. The P_K_ values were not altered throughout these estimates, as the effect was assumed to only be on the NALCN. This same procedure with simulations was repeated for the remaining strains.

**Figure 3:**
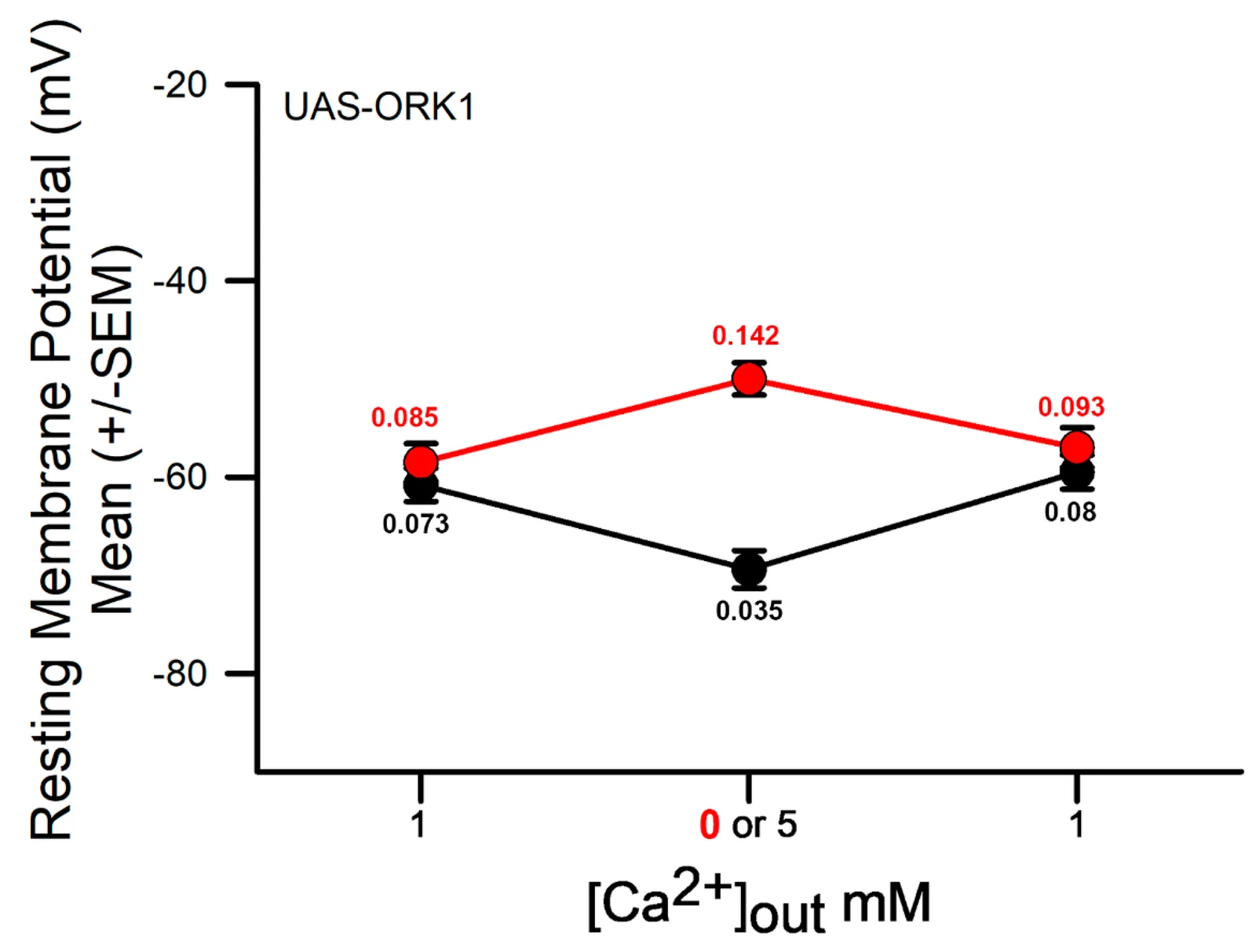
The effect on membrane potential for the parental strain UAS-ORK1 with changes from 1 mM to 0 or 5 mM and back to 1 mM [Ca^2+^]_o_. The average (±SEM) of the change for 10 individual preparations. *p* < 0.05, paired *t*-test, n = 10 for each of the paradigms 0 mM-1 mM or 0 mM to 5 mM. The estimated P_Na_/P_K_ values are presented for each condition. The P_K_ values were not altered in these estimates.

As for the parental strain UAS-ORK1-NC, the parental strain UAS-ORK1 showed very similar trends for the changes from 1 mM to 0 mM [Ca^2+^]_o_ and for 1 mM to 5 mM [Ca^2+^]_o_ with significant depolarization and hyperpolarization, respectively (Figure 3; *p* < 0.05, paired *t*-test, n = 10 for both paradigms).

**Figure 4:**
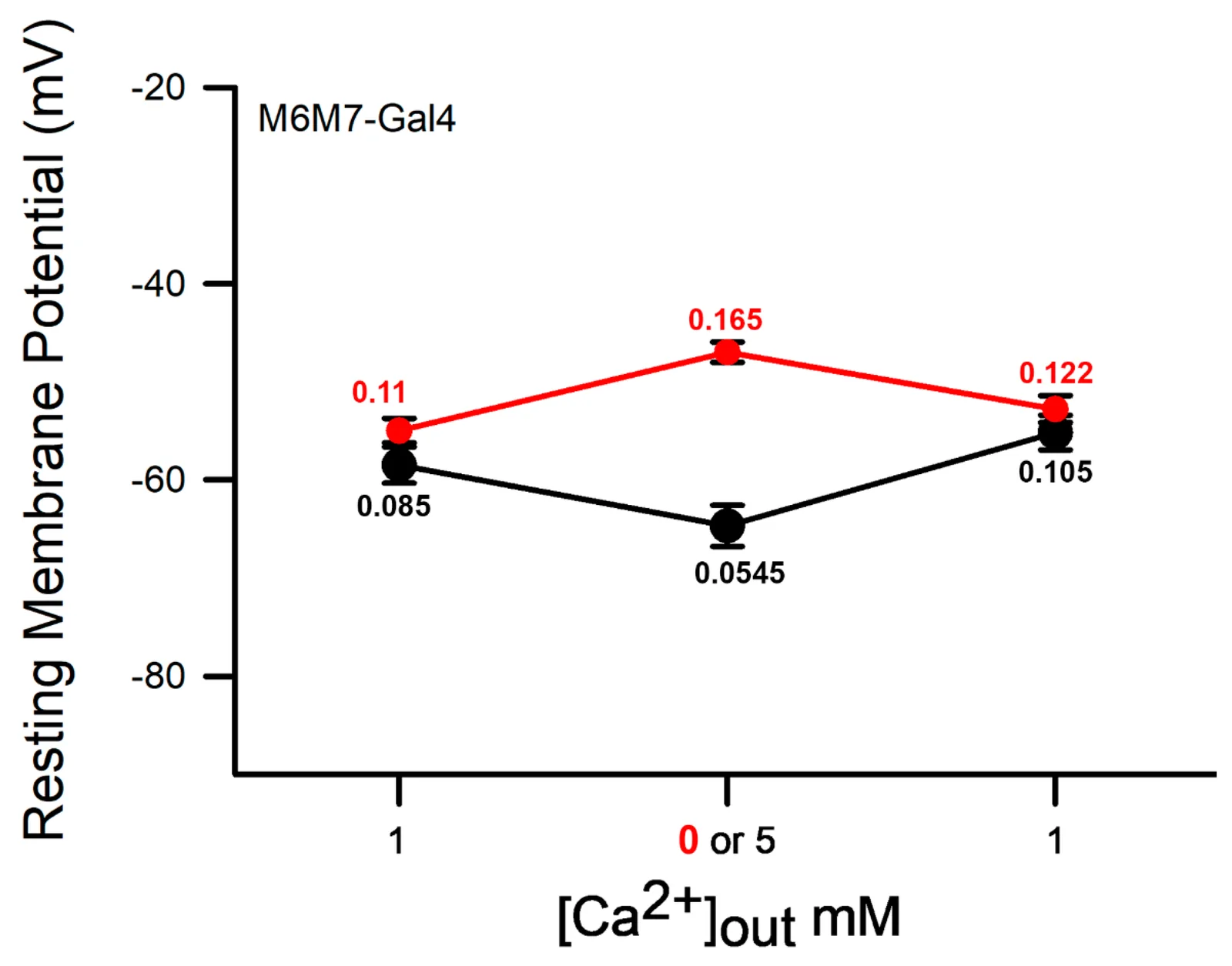
The effect on membrane potential for the parental strain M6M7-Gal4 with changes from 1 mM to only 0 or 5 mM and back to 1 mM [Ca^2+^]_o_. The average (±SEM) of the change for 10 individual preparations. *p* < 0.05, paired *t*-test, n = 10 for each of the paradigms 0 mM-1 mM or 0 mM to 5 mM. The estimated P_Na_/P_K_ values are presented for each condition. The P_K_ values were not altered in these estimates.

The other parental line M6M7-Gal4 also presented with the same trends for the changes in [Ca^2+^]_o_ from 1 mM to 0 mM and 1 mM to 5 mM, with significant depolarization and hyperpolarization, respectively (Figure 4; *p* < 0.05, paired *t*-test, n = 10 for both paradigms). This parental line also serves as a control for many of the F1 strains (M6M7>ORK1 NC; M6M7>ORK1; M6M7>NA-RNAi and M6M7>Mid1-RNAi) presented below, as they were crossed to males of this strain. The estimated P_Na_/P_K_ values indicate how much P_Na_ would need to change for each concentration of Ca^2+^ to either block or relieve the NALCN block. The P_K_ values were not altered throughout these estimates, as the effect was assumed to be only on the NALCN.

### 3.2. The Non-Conducting K2P Channel: Expressed in M6M7>ORK1-NC

**Figure 5:**
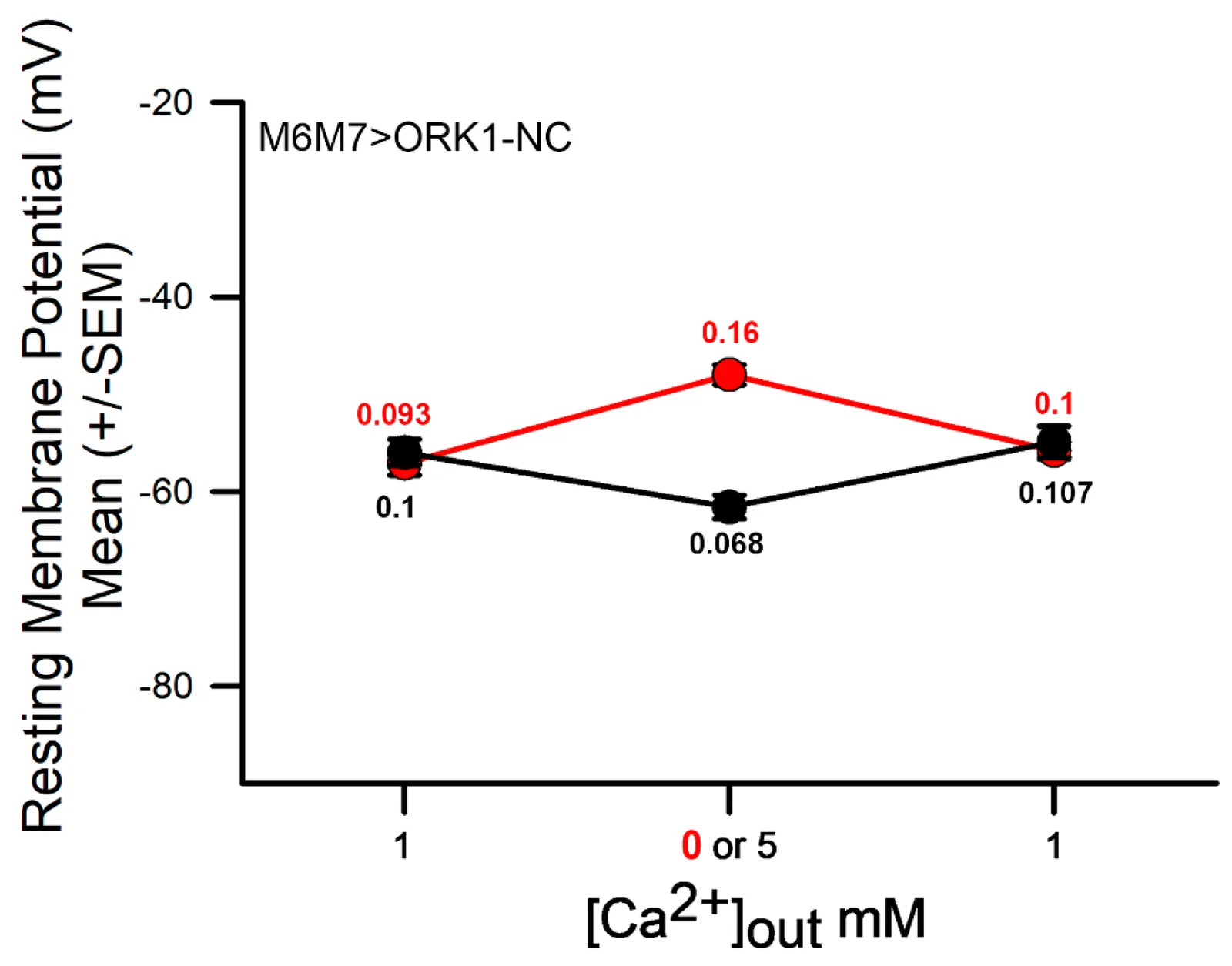
The effect on membrane potential for overexpressing the non-conductive ORK channel (M6M7>ORK1-NC) with changes from 1 mM to only 0 or 5 mM and back to 1 mM [Ca^2+^]_o_. The average (±SEM) of the change for 10 individual preparations. *p* < 0.05, paired *t*-test, n = 10 for each of the paradigms 0 mM-1 mM or 0 mM to 5 mM. We present the estimated P_Na_/P_K_ values for each condition. The P_K_ values were not altered in these estimates.

The effect of overexpression of the non-conducting K2P channel (i.e., ORK1-NC) in M6M7 was examined in M6 within segment 2 of the larvae, which presented a similar trend of the RP as the two parental lines (i.e., UAS-ORK1-NC and M6M7-Gal4). Bathing media of 1 to 5 mM [Ca^2+^]_o_ and 1 to 0 mM [Ca^2+^]_o_ produced significant hyperpolarization and depolarization, respectively (Figure 5; *p* < 0.05, paired *t*-test, n = 10 for both paradigms). In addition, the initial RP with 1 mM [Ca^2+^]_o_ was similar to the parental lines (compare Figure 2, Figure 3, Figure 4 and Figure 5). For the parental strains, the estimated P_Na_/P_K_ values indicate how much P_Na_ would need to change at each Ca^2+^ concentration to block or relieve the NALCN block. The P_K_ values were not altered throughout these estimates as the effect was assumed to be only on the NALCN, just as for the parental strains.

### 3.3. The Conducting K2P Channel: M6M7>ORK1

**Figure 6:**
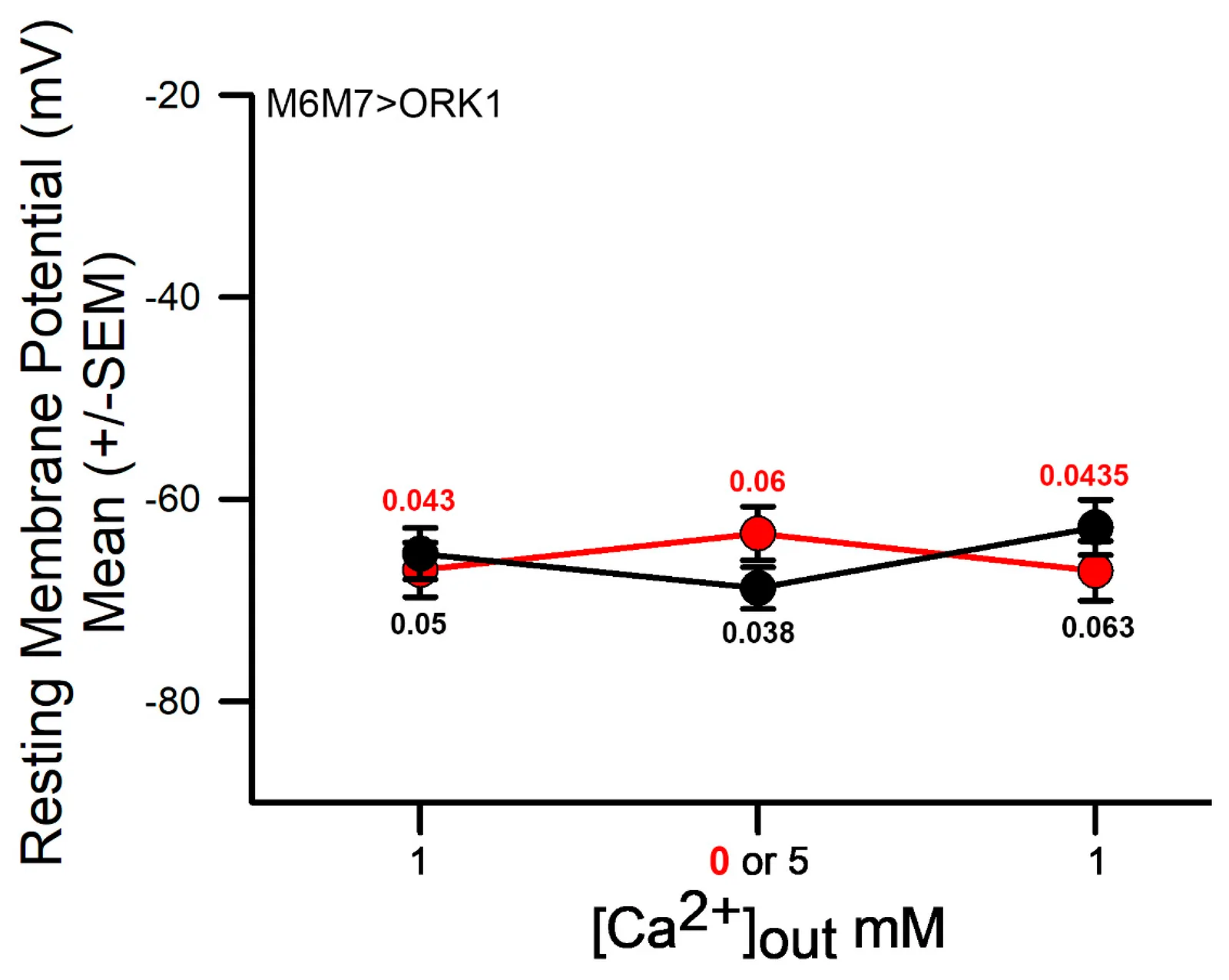
The effect on membrane potential for overexpressing the conductive ORK channel (M6M7>ORK1) with changes from 1 mM to only 0 or 5 mM and back to 1 mM [Ca^2+^]_o_. The average (±SEM) of the change for 10 individual preparations. *p* < 0.05, paired *t*-test, n = 10 for each of the paradigms 0 mM-1 mM or 0 mM to 5 mM. For the initial estimate of P_Na_/P_K_ at 1 mM [Ca^2+^]_o_, P_K_ was increased with P_Na_ the same as for the parentals at 1 mM [Ca^2+^]_o_. For the P_Na_/P_K_ values at each concentration of Ca^2+^, only P_Na_ was altered and not P_K_, from the initial values, as the changes in [Ca^2+^]_o_ were assumed to only be on the NALCN just as for the parental strains.

The effect of lower and higher [Ca^2+^]_o_ had a muted effect on the RP for the muscle fibers overexpressing the K2P channels (i.e., ORK1). Any change in driving the RP to be displaced by reducing (i.e., 0 mM [Ca^2+^]_o_) or promoting (5 mM [Ca^2+^]_o_) the block of the NALCN channels was attenuated by the strong K^+^ leak maintaining the RPs. However, altering the bathing media from 1 to 5 mM [Ca^2+^]_o_ and 1 to 0 mM [Ca^2+^]_o_ still produced a significant hyperpolarization and a depolarization, respectively (Figure 6; *p* < 0.05, paired *t*-test, n = 10 for both paradigms). Since this strain had an increased expression of ORK1 and a more negative RP, the estimated P_K_ was increased to match the membrane potential for the same P_Na_ initially as for the parental strains. The estimated P_Na_/P_K_ values indicate how much P_Na_ would need to change for each concentration of Ca^2+^ to either block or relieve the NALCN block from the initial values. The P_K_ values were not altered from the initial values for changes in [Ca^2+^]_o_, as the effect was assumed to be only on NALCN, as in the parental strains.

### 3.4. NALCN Channel: Parental UAS-NA and Parental UAS-MID1

**Figure 7:**
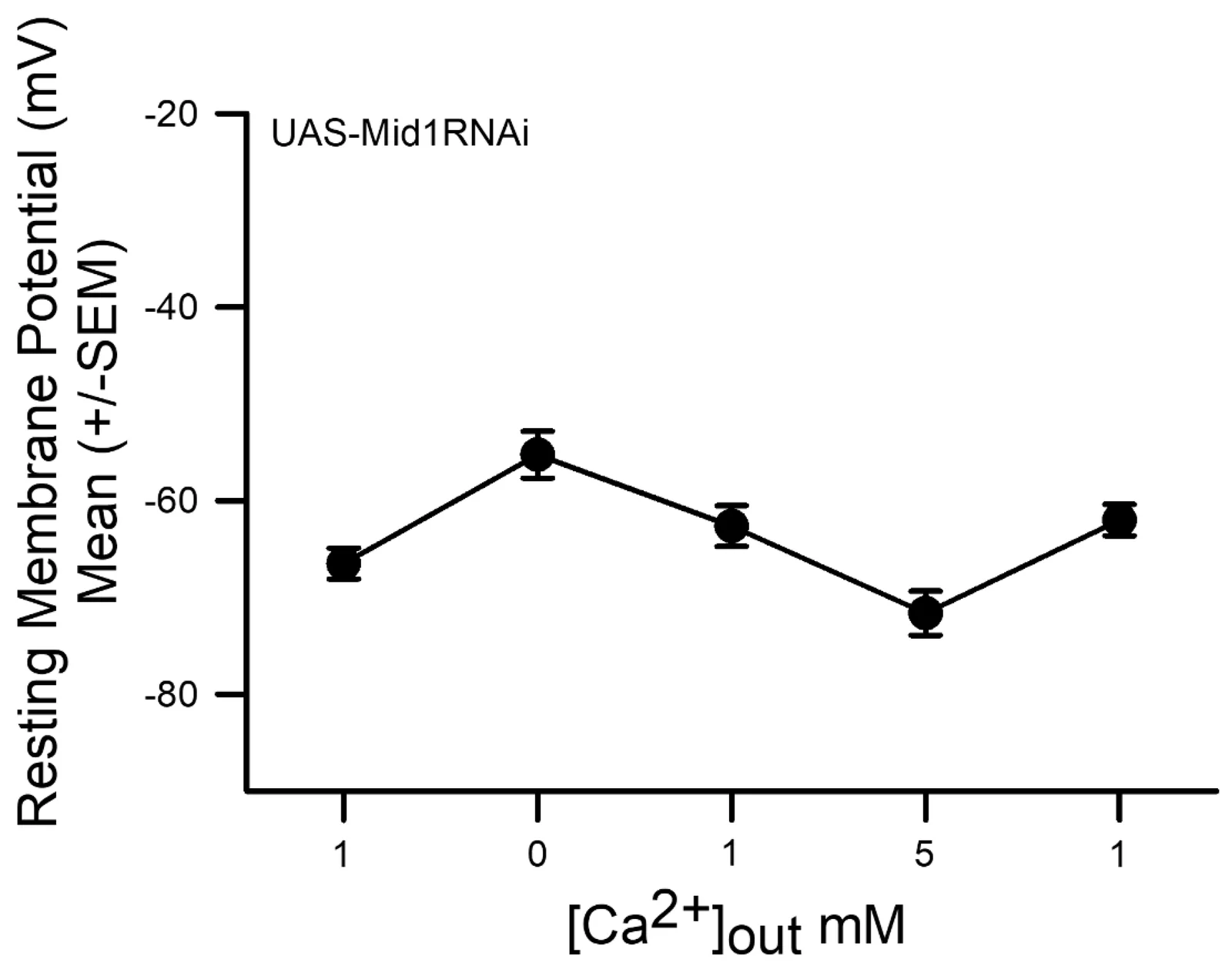
The effect on membrane potential for the parental UAS-Mid1-RNAi with changes from 1 mM to only 0 or 5 mM and back to 1 mM [Ca^2+^]_o_. The average (±SEM) of the change for eight individual preparations. *p* < 0.05, paired *t*-test, n = 8 for each of the paradigms (initial 1 mM to 0 mM and for the second exposure to 1 mM changed to 5 mM). The final 1 mM [Ca^2+^]_o_ was more depolarized than the initial 1 mM [Ca^2+^]_o_.

For the two RNAi strains (i.e., M6M7>NA-RNAi and M6M7>Mid1-RNAi), the parental strains (UAS-NA-RNAi and UAS-Mid1-RNAi) served as references. The results of altering [Ca^2+^]_o_ for M6M7-Gal4 are shown above in Section 3.1. The UAS-Mid1-RNAi strain was also examined with the full series [Ca^2+^]_o_ changes (1-0-1-5-1 mM [Ca^2+^]_o_). However, when the NMJ was exposed to 0 mM [Ca^2+^]_o_, the muscle again produced initial twitching and potential membrane damage, which made it difficult to obtain the same values of RP upon switching back to 1 mM [Ca^2+^]_o_ after the series of bathing changes. However, the same overall trends were observed as for the full series [Ca^2+^]_o_ changes (1-0-1-5-1 mM [Ca^2+^]_o_) for the parental strain of UAS-ORK1-NC. Each of the eight individual preparations showed the same trend, with depolarization in 0 mM and a subsequent hyperpolarization with 5 mM from the 1 mM Ca^2+^ exchanged after exposure to 0 mM (Figure 7; *p* < 0.05, paired *t*-test, n = 8).

Since the RPs did not appear to be stable over time with the serial changes in the [Ca^2+^]_o_ due to the initial muscle twitching with 0 mM [Ca^2+^]_o_, the single exposures to 0 mM or 5 mM of [Ca^2+^]_o_ were used as presented for the other strains. Perhaps if 5 mM [Ca^2+^]_o_ was used initially instead of 0 mM [Ca^2+^]_o_, a full series of changes could have been used; however, the responses appeared to be favorable with fewer changes of the bathing media, which decreased the likelihood of dislodging the microelectrode.

**Figure 8:**
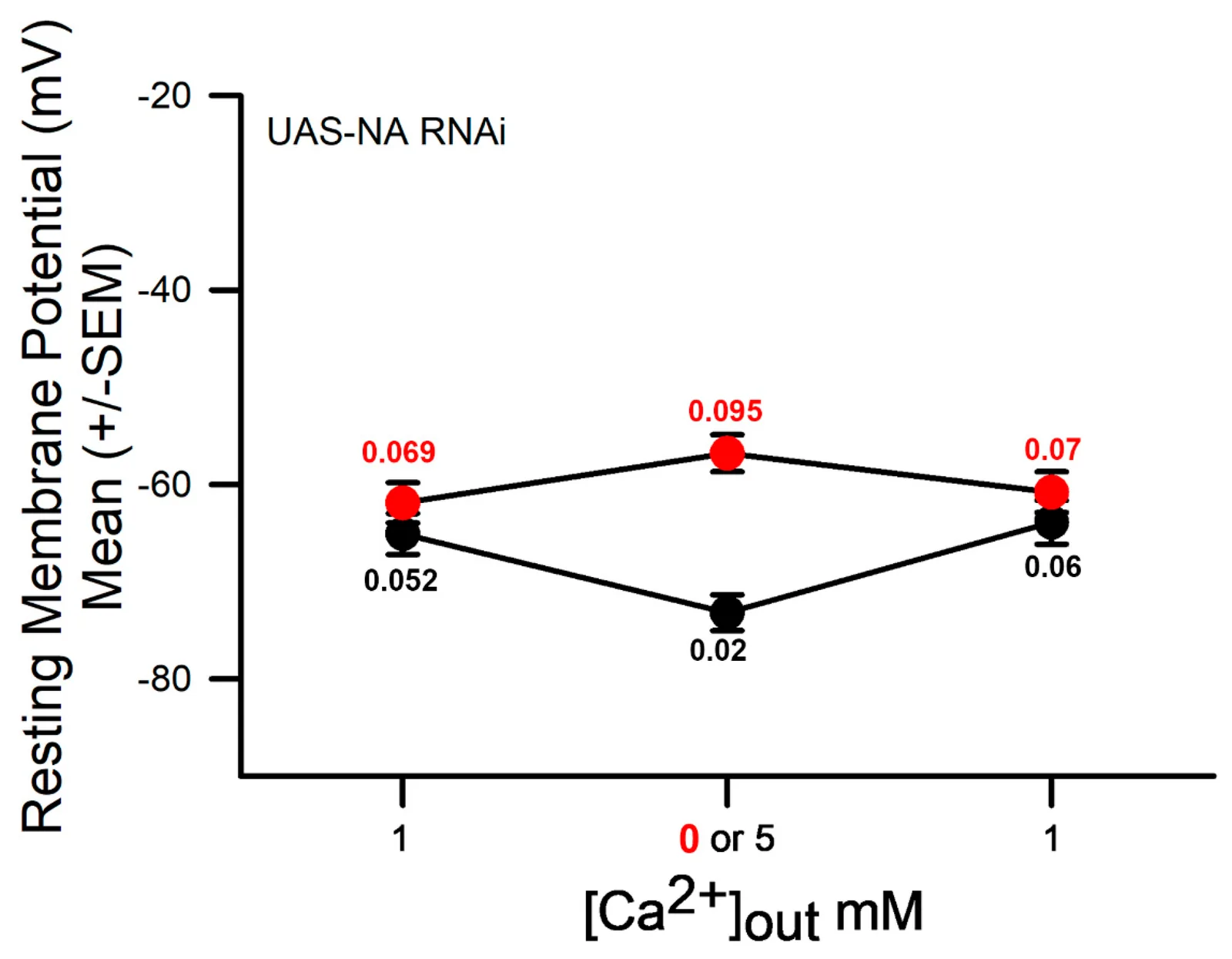
The effect on membrane potential for the parental strain (UAS-NA-RNAi) with changes from 1 mM to only 0 or 5 mM and back to 1 mM [Ca^2+^]_o_. The average (±SEM) of the change for 10 individual preparations. *p* < 0.05, paired *t*-test, n = 10 for each of the paradigms (0 mM-1 mM or 0 mM to 5 mM). The ratio of the P_Na_/P_K_ values was set to best provide a value close to RP for the initial values in 1 mM [Ca^2+^]_o_, and then, for the changes in [Ca^2+^]_o_, only the P_Na_ was altered to match the RP, as it was assumed to only affect NALCN permeability.

**Figure 9:**
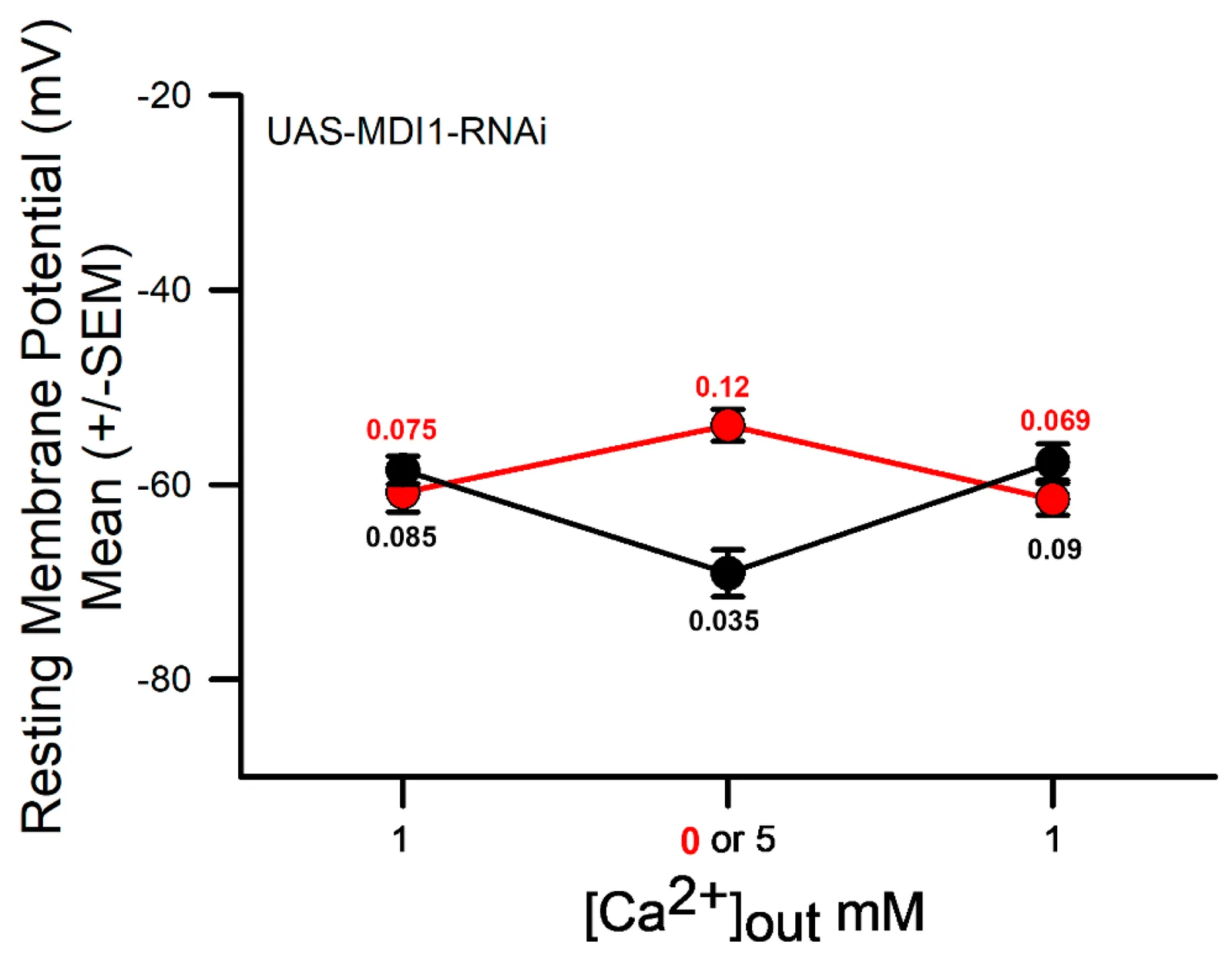
The effect on membrane potential for the parental strain (UAS-MID1-RNAi) with changes from 1 mM to only 0 or 5 mM and back to 1 mM [Ca^2+^]_o_. The average (±SEM) of the change for 10 individual preparations. *p* < 0.05, paired *t*-test, n = 10 for each of the paradigms (0 mM-1 mM or 0 mM to 5 mM). The ratio of the P_Na_/P_K_ values was set to best provide a value close to RP for the initial values in 1 mM [Ca^2+^]_o_, and then for the changes in the [Ca^2+^]_o_, only the P_Na_ was altered to match the RP, as it was assumed to only affect NALCN permeability.

Individual preparations of UAS-NA-RNAi and UAS-Mid1-RNAi were either examined with bath changes of 1 mM to 0 mM and back to 1 mM [Ca^2+^]_o_ or 1 to 5 mM and back to 1 mM [Ca^2+^]_o_. Both parental strains showed similar responses to the parental strains UAS-ORK1-NC and UAS-ORK1 above, with bathing media from 1 to 5 mM [Ca^2+^]_o_ and 1 to 0 mM [Ca^2+^]_o_ producing a significant hyperpolarization and a depolarization, respectively (Figure 8 UAS-NA-RNAi and Figure 9 UAS-MID1-RNAi; *p* < 0.05, paired *t*-test, n = 10 for both paradigms and for each strain). The ratio of the P_Na_/P_K_ values for both UAS-ORK1-NC and UAS-ORK1 was set to best provide a value close to RP for the initial values in 1 mM Ca^2+^]_o_ and then, for the changes in the [Ca^2+^]_o_, only the P_Na_ was altered to match the RP, as it was assumed to only affect NALCN permeability.

### 3.5. The Two Strains to Decrease the Influence of NALCN: M6M7>NA-RNAi and M6M7>Mid1-RNAi

**Figure 10:**
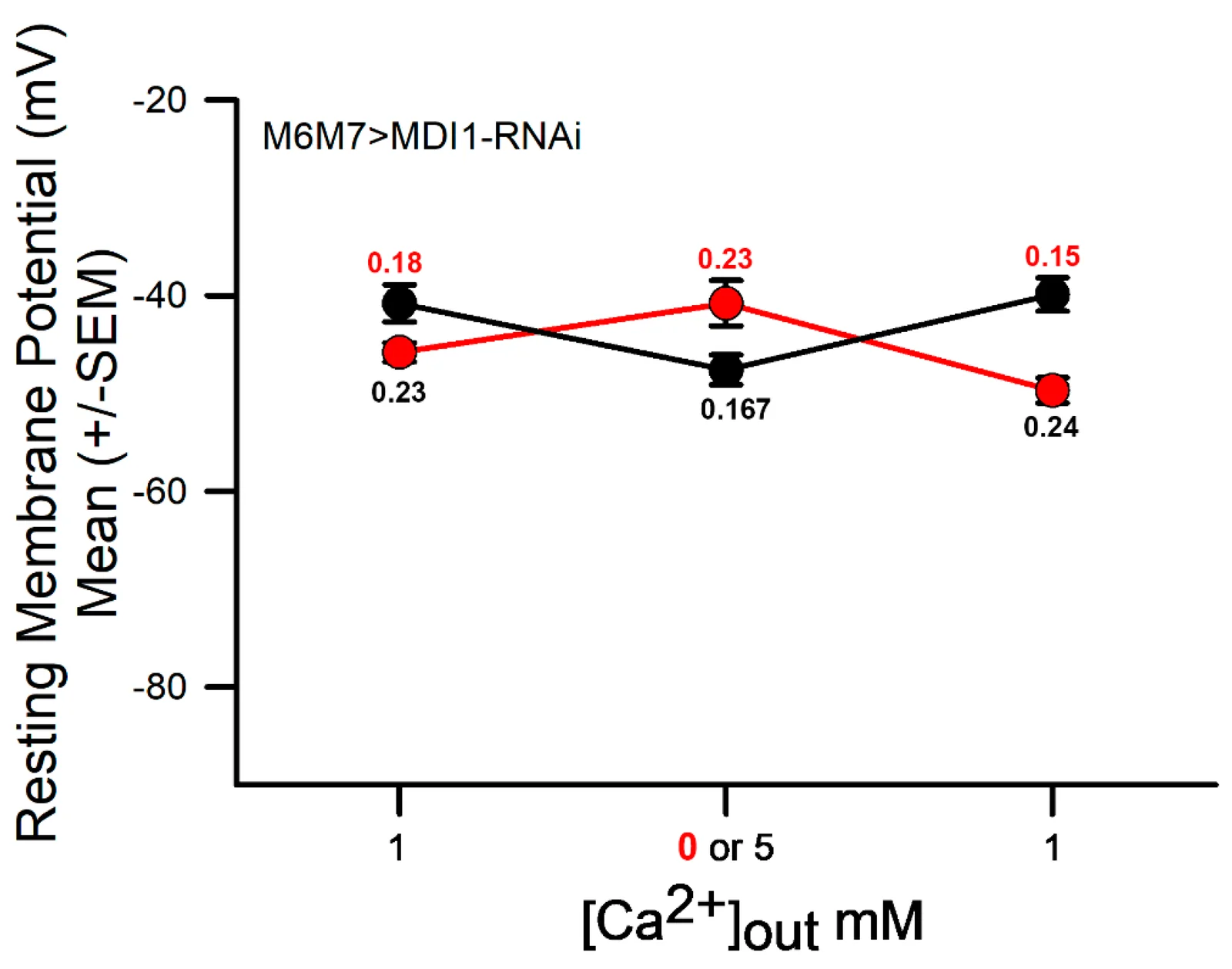
The effect on membrane potential for the M6M7>MID1-RNAi with changes from 1 mM to only 0 or 5 mM and back to 1 mM [Ca^2+^]_o_. The average (±SEM) of the change for 10 individual preparations. *p* < 0.05, paired *t*-test, n = 10 for each of the paradigms (0 mM-1 mM or 0 mM to 5 mM). The ratio of the P_Na_/P_K_ values was set to best provide a value close to RP for the initial values in 1 mM [Ca^2+^]_o_, and then, for the changes in [Ca^2+^]_o_, only the P_Na_ was altered to match the RP, as it was assumed to only affect NALCN permeability.

The effect of the strain (i.e., M6M7>Mid1-RNAi) in reducing the significance of the NALCN accessory protein Nif1 or Mid1 on Na^+^ leak through NALCN on the RP still showed similar trends to changes in [Ca^2+^]_o_. The changes of 1 to 5 mM [Ca^2+^]_o_ and 1 to 0 mM [Ca^2+^]_o_ still produced a significant hyperpolarization and a depolarization, respectively (Figure 10; *p* < 0.05, paired *t*-test, n = 10 for both paradigms).

The most unique feature of this strain was that the initial membrane potential with 1 mM [Ca^2+^]_o_ was depolarized compared to the parental controls UAS-MID1-RNAi and M6M7-Gal4 (ANOVA, *p* < 0.001, n = 20 for each strain). Thus, the ratio of the P_Na_/P_K_ values was set to best provide a value close to RP for the initial values in 1 mM [Ca^2+^]_o_, and then for the changes in [Ca^2+^]_o_, only the P_Na_ was altered to match the RP, as it was assumed to only affect NALCN permeability.

**Figure 11:**
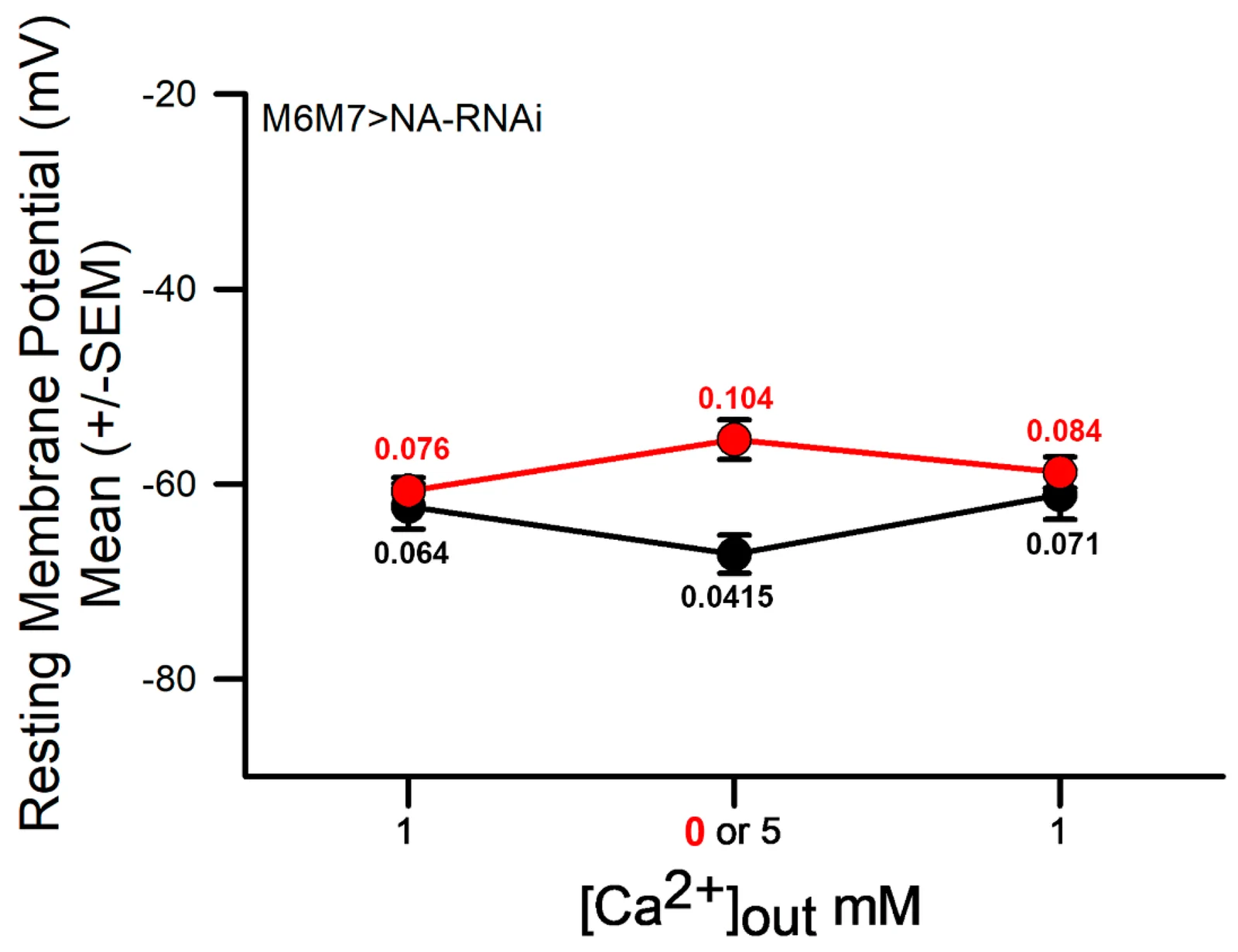
The effect on membrane potential for the M6M7>NA-RNAi with changes from 1 mM to only 0 or 5 mM and back to 1 mM [Ca^2+^]_o_. The average (±SEM) of the change for 10 individual preparations. *p* < 0.05, paired *t*-test, n = 10 for each of the paradigms (0 mM-1 mM or 0 mM to 5 mM). The ratio of the P_Na_/P_K_ values was set to best provide a value close to RP for the initial values in 1 mM [Ca^2+^]_o_, and then for the changes in the [Ca^2+^]_o_, only the P_Na_ was altered to match the RP, as it was assumed to only affect NALCN permeability.

The effect of the strain (i.e., M6M7>NA-RNAi), to reduce the significance of the NALCN channels directly on the RP, still presented similar trends to changes in [Ca^2+^]_o_ as for the M6M7>Mid1-RNAi strain. The changes of 1 to 5 mM [Ca^2+^]_o_ and 1 to 0 mM [Ca^2+^]_o_ produced a significant hyperpolarization and a depolarization, respectively (Figure 11; *p* < 0.05, paired *t*-test, n = 10 for both paradigms). However, for this strain, the initial membrane potential at 1 mM [Ca^2+^]_o_ was not significantly depolarized compared with the parental controls UAS-MID1-RNAi and M6M7-Gal4 (ANOVA, *p* > 0.05, n = 20 for each strain). Thus, the ratio of the P_Na_/P_K_ values was set to best provide a value close to RP for the initial values in 1 mM [Ca^2+^]_o_, which was close to the values for both UAS-ORK1-NC and UAS-ORK1. For changes in [Ca^2+^]_o_, only P_Na_ was altered to match the RP, as it was assumed to affect only NALCN permeability.

### 3.6. Comparisons of the Various Strains to the Effects of Varying [Ca^2+^]_o_

**Figure 12:**
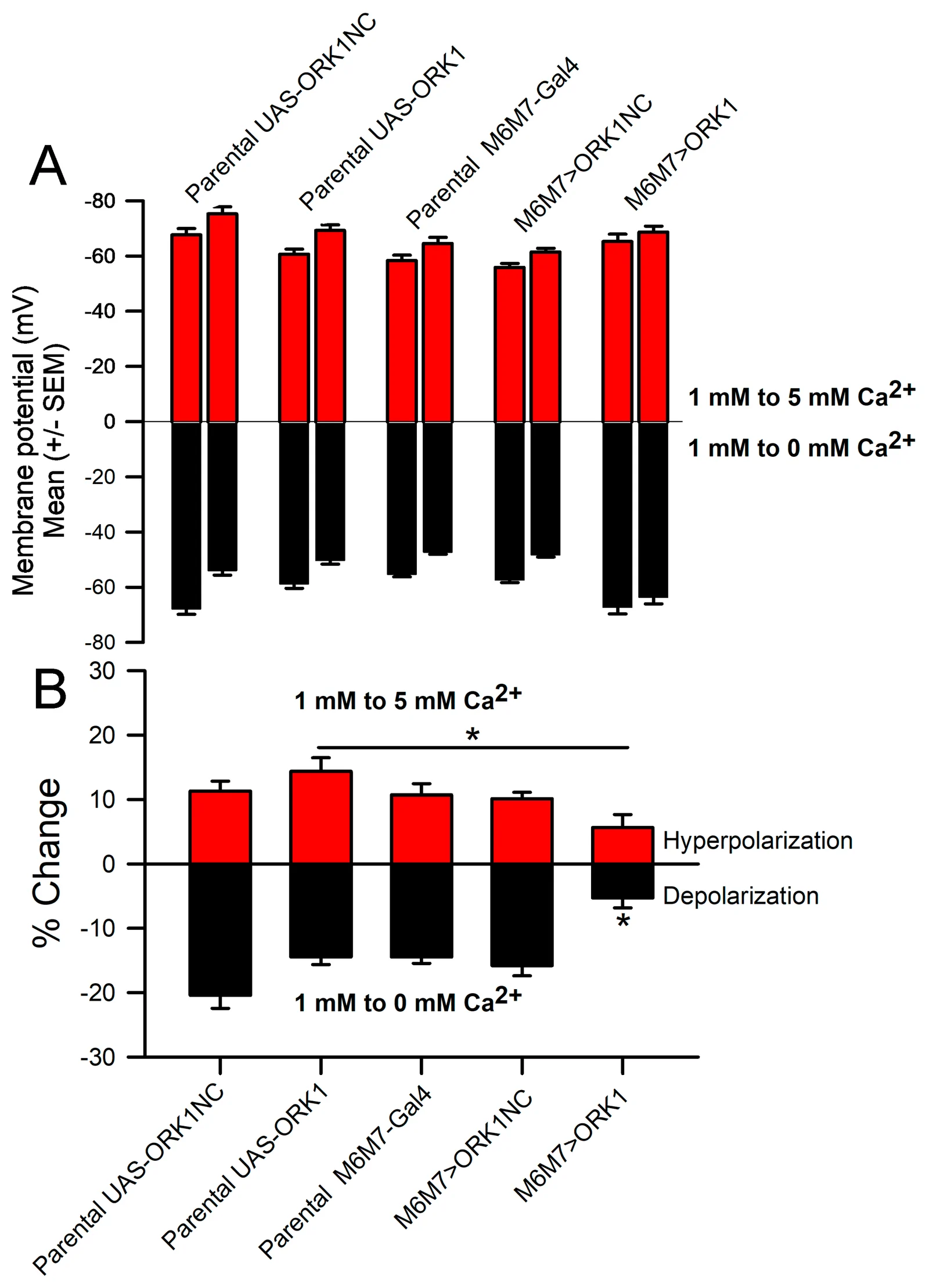
The membrane potentials and percent changes for each strain related to the ORK1 expression. (**A**) The means (±SEM) of the membrane potentials for 1 mM and 5 mM [Ca^2+^]_o_ (red) are shown for each strain, with the 1 mM being the first bar followed by 5 mM [Ca^2+^]_o_. The 1 mM and 0 mM [Ca^2+^]_o_ (back) with the 1 mM being the first bar followed by 0 mM [Ca^2+^]_o_. (**B**) The percent changes from 1 mM to 5 mM are shown in red and listed as hyperpolarized becoming larger/more negative), while the change to 0 mM is depolarized (less negative or a negative percent change) in membrane potential and is shown in black. There was a significant difference in each of these strains, UAS-ORK1-NC, UAS-ORK1, M6M7-Gal4, and M6M7>ORK1-NC to M6M7>ORK1 for the change from 1 mM to 0 mM [Ca^2+^]_o_ (ANOVA, * *p* < 0.001). The UAS-ORK1 strain and the M6M7>ORK1 strain were significantly different for the 1 mM to 5 mM [Ca^2+^]_o_ change (ANOVA, * *p* < 0.001).

For ease in direct comparisons of the membrane potentials (mean ± SEM) for the strains related to the ORK1 crosses, the values for 1 mM [Ca^2+^]_o_ to 0 mM or 5 mM [Ca^2+^]_o_ are shown side by side (Figure 12A) along with a percent change in the initial 1 mM [Ca^2+^]_o_ to 0 mM or 5 mM [Ca^2+^]_o_ (Figure 12B). The percentage changes for each strain to 5 mM are shown in red and listed as hyperpolarized becoming larger/more negative), while the change to 0 mM is depolarized (less negative or a negative percent change) in membrane potential. The same color coding was maintained for the means of the membrane potentials as shown in Figure 12A. There was a significant difference in each of these strains, UAS-ORK1-NC, UAS-ORK1, M6M7-Gal4, and M6M7>ORK1-NC to M6M7>ORK1 for the change from 1 mM to 0 mM [Ca^2+^]_o_ (Shapiro–Wilk Normality Test; Brown–Forsythe Equal Variance Test; ANOVA Holm–Sidak with *p* < 0.001). However, only the UAS-ORK1 strain differed significantly from the M6M7>ORK1 strain for the 1 mM to 5 mM [Ca^2+^]_o_ change (Shapiro–Wilk Normality Test; Brown–Forsythe Equal Variance Test; ANOVA Holm-Sidak with *p* < 0.001).

**Figure 13:**
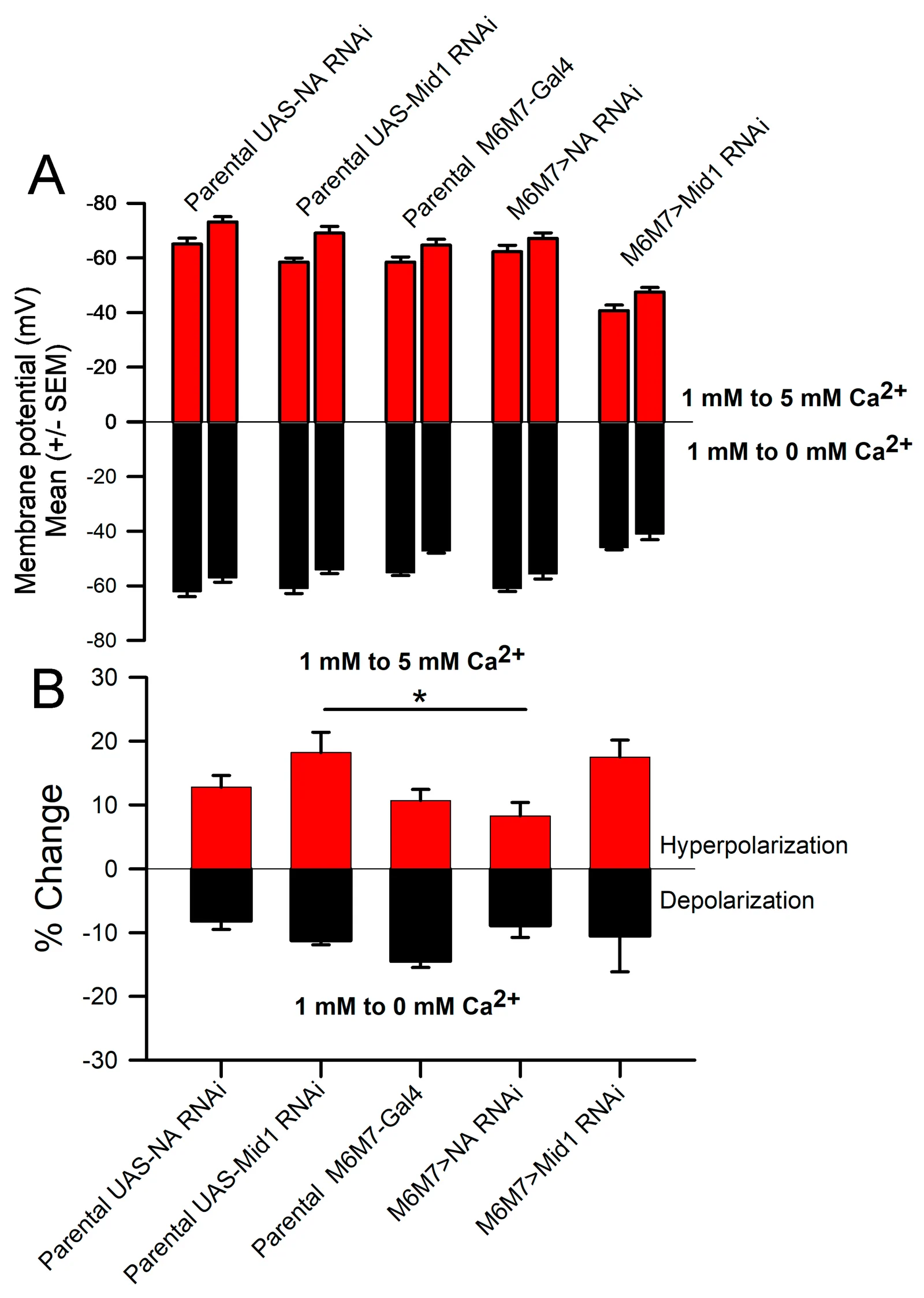
Membrane potentials and percent changes for each strain related to the RNAi and parental strains for NALCN with varying [Ca^2+^]_o_. (**A**) The means (±SEM) of the membrane potentials for 1 mM and 5 mM [Ca^2+^]_o_ (red) are shown for each strain, with 1 mM being the first bar followed by 5 mM [Ca^2+^]_o_. The 1 mM and 0 mM [Ca^2+^]_o_ (back) with the 1 mM being the first bar followed by 0 mM [Ca^2+^]_o_. (**B**) The percent changes from 1 mM to 5 mM are shown in red and listed as hyperpolarizing (becoming larger/more negative), while the change to 0 mM, as depolarizing (less negative or a negative percent change) in membrane potential, is shown in black. There was only a significant difference in strains UAS-MID1-RNAi and M6M7>NA-RNAi for the change from 1 mM to 5 mM [Ca^2+^]_o_ (ANOVA, * *p* < 0.05). The other strains did not differ significantly between 1 mM and 5 mM [Ca^2+^]_o_ or between 1 mM and 0 mM [Ca^2+^]_o_.

For ease of direct comparison of the membrane potentials (mean ± SEM) for the strains related to the RNAi and parental strains for the NALCN, the values for 1 mM [Ca^2+^]_o_ to 0 mM or 5 mM [Ca^2+^]_o_ are shown side by side (Figure 13A) along with a percent change in the initial 1 mM [Ca^2+^]_o_ to 0 mM or 5 mM [Ca^2+^]_o_ (Figure 13B). The % changes for each strain to 5 mM are shown in red and listed as hyperpolarized (more negative), while the change to 0 mM is depolarized (less negative or a negative percent change) in membrane potential. The same color coding was used for the mean membrane potentials, as shown in Figure 13A. There was only a significant difference in strains UAS-MID1-RNAi and M6M7>NA-RNAi for the change from 1 mM to 5 mM [Ca^2+^]_o_ (Shapiro–Wilk Normality Test; Brown–Forsythe Equal Variance Test; ANOVA Holm-Sidak with *p* < 0.05). The other strains did not differ significantly between 1 mM and 5 mM [Ca^2+^]_o_ or between 1 mM and 0 mM [Ca^2+^]_o_.

## 4. Discussion

This investigation revealed that using the *Drosophila* larval body wall muscles as a model, increasing [Ca^2+^]_o_ hyperpolarized the membrane potential and that decreasing [Ca^2+^]_o_ resulted in depolarization of the resting membrane potential (Figure 12 and Figure 13). Given that it is generally accepted that a likely mechanism is due to Ca^2+^ ions being able to block the NALCN, these findings were expected [3,19,20,21,40]. Two different approaches in reducing the ability of NALCNs to have an effect were examined. The function of the channels was attempted to be dampened by reducing the effect of the accessory protein to the channel by using a *Drosophila* form of RNAi for MID1 protein. This approach showed no differences compared to the control parental strains for the changes induced by varying [Ca^2+^]_o_. The second approach used *Drosophila* RNAi for NALCN expressed in select skeletal muscles. This was only a slight reduction, although significant, in the effects of raised [Ca^2+^]_o_ compared to the control parental strains. Lowering [Ca^2+^]_o_ resulted in depolarization equal to that of the parental strains UAS-NA-RNAi. To ensure that UAS-GAL4 expression was functioning for the selected muscles, and to examine the effect of altering [Ca^2+^]_o_ on muscles overexpressing the ORK1 potassium leak channels (i.e., K2P), the same series of experiments were conducted in varying [Ca^2+^]_o_ while monitoring the membrane potential strain. As an extra check for examining if an increasing expression of proteins (i.e., ORK or the forms of RNAi) may account for non-targeted issues, a missense in the ORK gene was expressed, which expresses a non-conductive K2P protein. This strain showed no differences compared to parental strains of ORK or of or NA-RNAi and MID1-RNAi.

The initial approach of changing out the salines to different [Ca^2+^]_o_ as a series was not feasible due to the muscle contractions induced by exposure to saline at 0 mM [Ca^2+^]_o_. The membrane had probably become slightly damaged by the microelectrode during the contractions, as even returning to the initial saline at 1 mM [Ca^2+^]_o_, the membrane potentials were still slightly depolarized. Perhaps removing the electrode and penetrating the fiber again would be an option, but the fiber would still be contracting repetitively while bathed in 0 mM [Ca^2+^]_o_, making it difficult to obtain a recording without damaging the membrane. To reduce the time required to maintain an intracellular recording, we preferred using different preparations for each [Ca^2+^]_o_ condition, which yielded more reliable readings.

Such strong hyperpolarization in the strain overexpressing the K2P channels overrode the effect of Ca^2+^ blocking the NALCN, or even relieved the Ca^2+^ block. Since the membrane potential only reached an average of approximately −70 mV with the overexpression of ORK-K2P channels, it is likely that the membrane was still permeable to some Na^+^, as it is estimated that the E_K_ is around −90 mV for adult *Drosophila* muscles [41,42] and maybe even as large as −100 mV for these larval muscles [18]. Or perhaps the pumps and ion exchangers tried to help maintain a homeostatic membrane potential close to the normal values. Despite not adding any CaCl_2_ to the saline, for the 0 mM trials, the membrane potential still did not depolarize to what would normally be obtained at the peak of an evoked EJP for the combined recruitment of Ia and Ib motor nerve terminals innervating the muscle fiber [43]. Postsynaptic glutamate receptors at NMJ synapses are ionotropic and allow Na^+^ influx with some K^+^ efflux [44]. This indicates that relieving the NALCN block by reducing [Ca^2+^]_o_ did not even allow as much Na^+^ to influx as would occur during an evoked synaptic event.

It is feasible that Mg^2+^ has taken on the role of blocking the NALCN, particularly in the absence of Ca^2+^ [20]. Only a 10 mM concentration of MgCl_2_ was used in these experiments, but considering that only 0 to 5 mM of CaCl_2_ resulted in significant effects on membrane potential, perhaps future investigations could conduct similar experiments in the absence of, or at higher concentrations of, MgCl_2_ and CaCl_2_ to examine if the membrane potential was more drastically affected with and without functional NALCNs.

Considering that overexpression of the ORK-K2P channels dampened the effects of changes in [Ca^2+^]_o_, the opposite may also occur with a lower expression of K2P channels, allowing the membrane potential to be more sensitive to the fluctuations in [Ca^2+^]_o_ or even, for that matter, [Mg^2+^]_o_. Currently, we are reducing the function of a fraction of the K2P channels and addressing this topic, but again the problem exists: if the muscle depolarizes, it starts to contract, making it difficult to obtain stable membrane potentials.

The estimated P_Na_/P_K_ ratios provide a level of understanding of the degree of changes likely in P_Na_ as [Ca^2+^]_o_ is changed, assuming most of the shift in membrane potential is due to the NALCN. The generalized GHK equation for estimating membrane potential does not take into consideration separately the electrogenic nature of the Na-K ATPase pump or Ca^2+^-pump (PMCA) or exchangers such as the NCX, but it does allow some index to compare to when one variable is being changed experimentally, such as [Ca^2+^]_o_. In this report, it is assumed that only P_Na_ is altered as a single variable, which is not likely, but there is not enough information yet to know how other factors might have a role. Generally, the initial P_Na_/P_K value_ for all strains at 1 mM [Ca^2+^]_o_, except the M6M7>ORK1 strain, was similar and increased at 0 mM [Ca^2+^]_o_ and decreased at 5 mM [Ca^2+^]_o_. The M6M7>ORK1 strain started off with a low P_Na_/P_K_ and was increased with 0 mM [Ca^2+^]_o_ and decreased with 5 mM [Ca^2+^]_o_. This was less of a change as compared to the other strains. This again was due to the large P_K_ masking any effect on P_Na_ by changes in [Ca^2+^]_o_.

The role of the accessory proteins with NALCN is not fully established, but it was shown that reducing MID1 by RNAi perturbed the function of the NALCN channels and decreased the expression of the channel [25]. However, this was not previously examined in skeletal muscles of *Drosophila* larvae. Likewise, the use of RNAi for protein expression of the *na* gene in *Drosophila* previously showed that expression of NALCN was decreased [8]. Future studies could examine, with mRNA sequencing, if other such proteins may also be affected by these RNAi approaches. In fact, it is possible that, with a reduction of MID1, additional accessory proteins were upregulated, maybe even compensating for the loss of MID1.

Even though the strain (i.e., BG487-Gal4) produces high expression in muscles M6M7 within segments 1 and 2 and a reduced expression from an anteroposterior pattern in the M6M7 segmental muscles [34,35], the larvae were still very active with crawling movements for the M6M7>NA-RNAi strain. However, there were a number of larvae that died in various trials in all larval stages (1st, 2nd, and 3rd) when obtaining the F1 generation for the M6M7>MID1-RNAi strain. MID1 may also have roles beyond being an accessory protein for NALCN. The early 3rd instar larvae in both F1 RNAi strains used in this investigation had stable resting membrane potentials for M6 in segment 2 when initially measured; although the M6M7>MID1-RNAi strain had a significantly depolarized level (~−40 to−45 mV) as compared to the M6M7>NA-RNAi or any of the other parental strains (~−55 to−60 mV; *p* < 0.05, ANOVA). The notable baseline depolarization in the M6M7>MID1-RNAi strain is a significant finding that may indicate additional physiological effects of MID1 depletion. The future evaluation of the Ca^2+^ responses would provide information on the baseline difference. Future investigations with higher and lower concentrations of both Mg^2+^ and Ca^2+^ would help address this matter.

Given that there is a retrograde communication from the muscle to motor neurons throughout development which maintains synaptic function [45,46,47,48], it is not known how an altered chronic membrane potential of the postsynaptic target might impact the innervating motor neurons and, secondarily, the target’s response. This could even have a spiraling effect over larval development. Much remains to be learned about the impact of retrograde signaling on overall synaptic physiology, as well as its subtle effects on pre- and postsynaptic cells.

## Data Availability

The original contributions presented in this study are included in the article. Further inquiries can be directed to the corresponding author.

## Appendix A

The parameters used for coding the GHK estimations (reproduced from [36]).

For running the GHK Equation to estimate equilibrium potentials, the GitHub code was used https://github.com/ywkim17/Educational_Book/blob/main/GHK.py (accessed on 10 August 2026). The code named “GHK.py” was run on the VSCode software.

import math

def ghk(R, T, F, P_K, K_in, K_out, P_Na, Na_in, Na_out, P_Cl, Cl_in, Cl_out):

&quot;&quot;&quot;

Computes membrane potential (Vm) in millivolts using the Goldman-Hodgkin-Katz equation.

Parameters:

R: float—Universal gas constant (J/(mol·K))

T: float—Temperature in Kelvin (K)

F: float—Faraday's constant (C/mol)

P_K: float—Permeability of potassium

K_in: float—Intracellular potassium concentration (mM)

K_out: float—Extracellular potassium concentration (mM)

P_Na: float—Permeability of sodium

Na_in: float—Intracellular sodium concentration (mM)

Na_out: float—Extracellular sodium concentration (mM)

P_Cl: float—Permeability of chloride

Cl_in: float—Intracellular chloride concentration (mM)

Cl_out: float—Extracellular chloride concentration (mM)

P_Ca: float—Permeability of calcium

Ca_in: float—Intracellular calcium concentration (mM)

Ca_out: float—Extracellular calcium concentration (mM)

Returns:

Vm (float): Membrane potential in millivolts (mV)

&quot;&quot;&quot;

# Calculate RT/F in millivolts

V_T = (R * T)/F * 1000 # Convert to mV

numerator = (P_K * K_out + P_Na * Na_out + P_Cl * Cl_in)

denominator = (P_K * K_in + P_Na * Na_in + P_Cl * Cl_out)

if denominator == 0:

raise ValueError(&quot;Denominator is zero; check input values.&quot;)

Vm = V_T * math.log(numerator/denominator)

return Vm

# Parameter Inputs

R = 8.314 #Constant Value

T = 294.15

F = 96,485 #Constant Value

P_K, K_in, K_out = 1.3, 140, 220

P_Na, Na_in, Na_out = 0.001, 15, 10

P_Cl, Cl_in, Cl_out = 0.8, 10, 110

# Compute Membrane Potential

Vm = ghk(R, T, F, P_K, K_in, K_out, P_Na, Na_in, Na_out, P_Cl, Cl_in, Cl_out)

print(f&quot;\nMembrane potential: {Vm:.2f} mV&quot;)

The initial values for starting the simulations for the larval Drosophila muscle were as shown below.

The saline used for larval *Drosophila* physiological measures is: (in mmol/L) 70 NaCl, 5 KCl, 20

MgCl_2_, 10 NaHCO_3_, 1 CaCl_2_, 5 trehalose, 115 sucrose, 25 N,N-bis(2-hydroxyethyl)-2-

aminoethane sulfonic acid (BES) and pH at 7.2.

[K^+^]_i_ = 190 mM (estimated for *Drosophila* muscle)

[K^+^]_o_ = 5 mM (Saline)

[Na^+^]_i_ = 12 mM (assume for *Drosophila* muscle)

[Na^+^]_o_ = 80 mM (Saline)

[Cl^−^]_i_ = 12 mM (assume for muscle)

[Cl^−^]_o_ = 91 mM

pK = 1 (assume for muscle)

pNa = 0.001 (assume for muscle)

pCl = 0.01 (assume for muscle)
